# Case reports of atrial and pericardial rupture from blunt cardiac trauma

**DOI:** 10.1186/s13019-018-0753-2

**Published:** 2018-06-19

**Authors:** D. Baldwin, K. L. Chow, H. Mashbari, E. Omi, J. K. Lee

**Affiliations:** 1 Department of Surgery, Division of Surgical Critical Care, University of Illinois at Chicago, 1740 W Taylor St, Chicago, IL 60612 USA; 2 Department of Trauma, Division of Trauma /Surgical Critical Care, Advocate Christ Medical Center, Oak Lawn, IL USA

**Keywords:** Atrial rupture, Pericardial rupture, Blunt cardiac trauma

## Abstract

**Background:**

Blunt cardiac trauma is diagnosed in less than 10% of trauma patients and covers the range of severity from clinically insignificant myocardial contusions to lethal multi-chamber cardiac rupture. The most common mechanisms of injury include: motor vehicle collisions (MVC), pedestrians struck by motor vehicles and falls from significant heights. A severe complication from blunt cardiac trauma is cardiac chamber rupture with pericardial tear. It is an exceedingly rare diagnosis. A retrospective review identified only 0.002% of all trauma patients presented with this condition. Most patients with atrial rupture do not survive transport to the hospital and upon arrival diagnosis remains difficult.

**Case presentation:**

We present two cases of atrial and pericardial rupture. The first case is a 33-year-old female involved in a MVC, who presented unresponsive, hypotensive and tachycardic. A left sided hemothorax was diagnosed and a chest tube placed with 1200 mL of bloody output. The patient was taken to the OR emergently. Intraoperatively, a laceration in the right pericardium and a 3 cm defect in the anterior, right atrium were identified. Despite measures to control hemorrhage and resuscitate the patient, the patient did not survive.

The second case is a 58-year-old male involved in a high-speed MVC. Similar to the first case, the patient presented unresponsive, hypotensive and tachycardic with a left sided hemothorax. A chest tube was placed with 900 mL of bloody output. Based on the output and ongoing resuscitation requirements, the patient was taken to the OR. Intraoperatively, a 15 cm anterior pericardial laceration was identified. Through the defect, there was brisk bleeding from a 1 cm laceration on the left atrial appendage. The injury was debrided and repaired using a running 3–0 polypropylene suture over a Satinsky clamp. The patient eventually recovered and was discharged home.

**Conclusions:**

We present two cases of uncontained atrial and pericardial rupture from blunt cardiac trauma. Contained ruptures with an intact pericardium present as a cardiac tamponade while uncontained ruptures present with hemomediastinum or hemothorax. A high degree of suspicion is required to rapidly diagnose and perform the cardiorrhaphy to offer the best chance at survival.

## Background

Blunt cardiac trauma is diagnosed in less than 10% of trauma patients and covers the range of severity from clinically insignificant myocardial contusions to lethal multi-chamber cardiac rupture [1]. The most common mechanisms of injury include: motor vehicle collisions (MVC) (50%), pedestrians struck by motor vehicles (35%), motorcycle crashes (9%), and falls from significant heights [2]. In one autopsy study, cardiac injury was identified in 11.9% of over 1600 fatalities from blunt trauma and was either the only cause of death or contributed to the fatal outcome in 45 to 76% of those cases [2–4]. Although blunt traumatic atrial tears have been reported to have a better prognosis than ventricular ruptures, these injuries remain rapidly fatal and require a high index of suspicion and emergent operative repair [4–6].

Blunt traumatic rupture of the heart and pericardium, rarely diagnosed preoperatively, carries a high mortality rate. The National Trauma Data Bank reports that chamber rupture represents 0.041% of all trauma cases and has an overall mortality rate of 89.2% [7]. A retrospective review of more than 20,000 patients admitted from 1979 to 1989 to a single Level I trauma center identified 59 (0.002%) patients requiring emergency surgery for this condition. The overall mortality rate was 76% (45 patients), and only 52% for those with vital signs on admission [8]. A similar study showed 11 patients out of 58,304 (0.0002%) trauma activations were diagnosed with blunt cardiac rupture over a five year period [9]. Finally, in a review of 4169 victims of traffic collisions fatally injured between 1972 to 1985, chest injuries were recorded as the main cause of death in 1121 victims. Despite wearing a seatbelt, 75 out of 207 patients were found to have cardiac rupture on autopsy. In frontal impact collisions, the mechanism leading to cardiac rupture is usually a crushing force against the steering wheel, with increasing severity depending on vehicle mass and velocity [10].

Most patients with atrial rupture do not survive transport to the hospital. In those who present to hospitals, diagnosis remains difficult. Traditionally, blunt cardiac rupture with an intact pericardium presents with signs and symptoms of cardiac tamponade. Whereas, cardiac rupture with torn pericardium present with hemomediastinum or hemothorax [11]. When evaluating a hemothorax from blunt trauma, atrial rupture with combined pericardial rupture remains low on the differential but cannot be neglected. We present two cases to illustrate this rare diagnosis.

## Case Report One

**Fig. 1:**
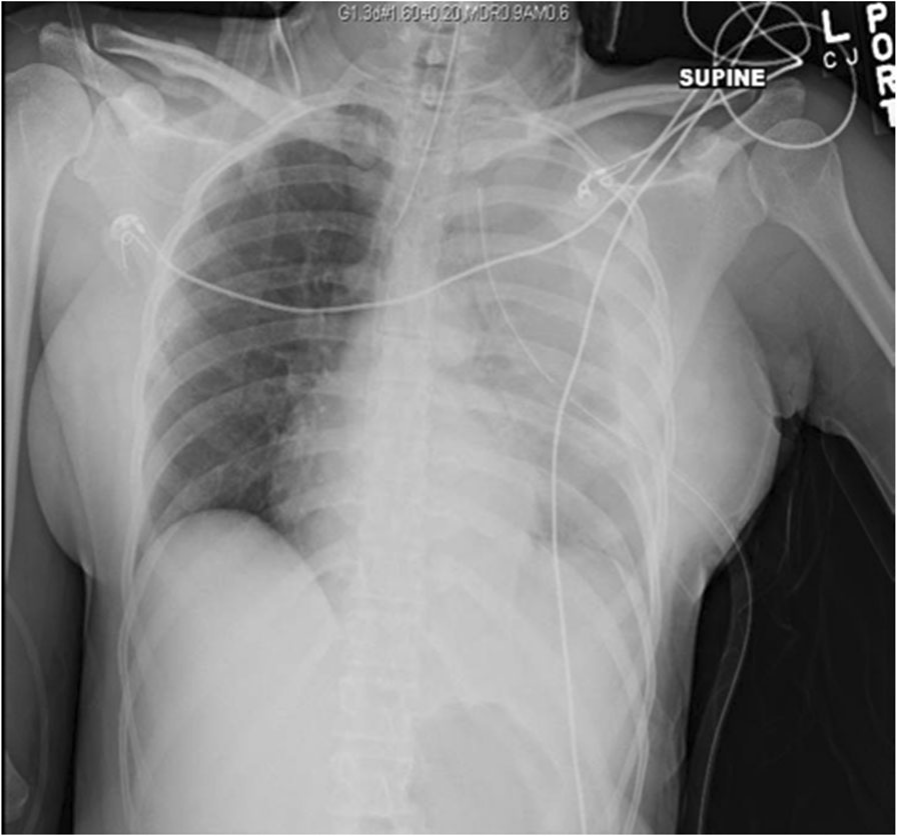
Case 1: Left sided hemothorax

We present the case of a 33-year-old female who was an unrestrained driver in a MVC with major front-end damage where airbags were deployed. She arrived to Advocate Christ Medical Center (a high volume, academic, level 1 trauma center) unresponsive with a Glasgow Coma Scale (GCS) of 5 and was intubated for airway protection. Breath sounds were noted to be present bilaterally. She was tachycardic and hypotensive with a heart rate of 143 and blood pressure of 71/46. A focused assessment with sonography for trauma (FAST) demonstrated no evidence of intra-abdominal or pericardial fluid. A left sided hemothorax was present on chest x-ray, and a chest tube was placed with 1200 mL of bloody output initially (See Fig. 1). At this point, the decision was made to take patient emergently to the operating room (OR) for exploration.

**Fig. 2:**
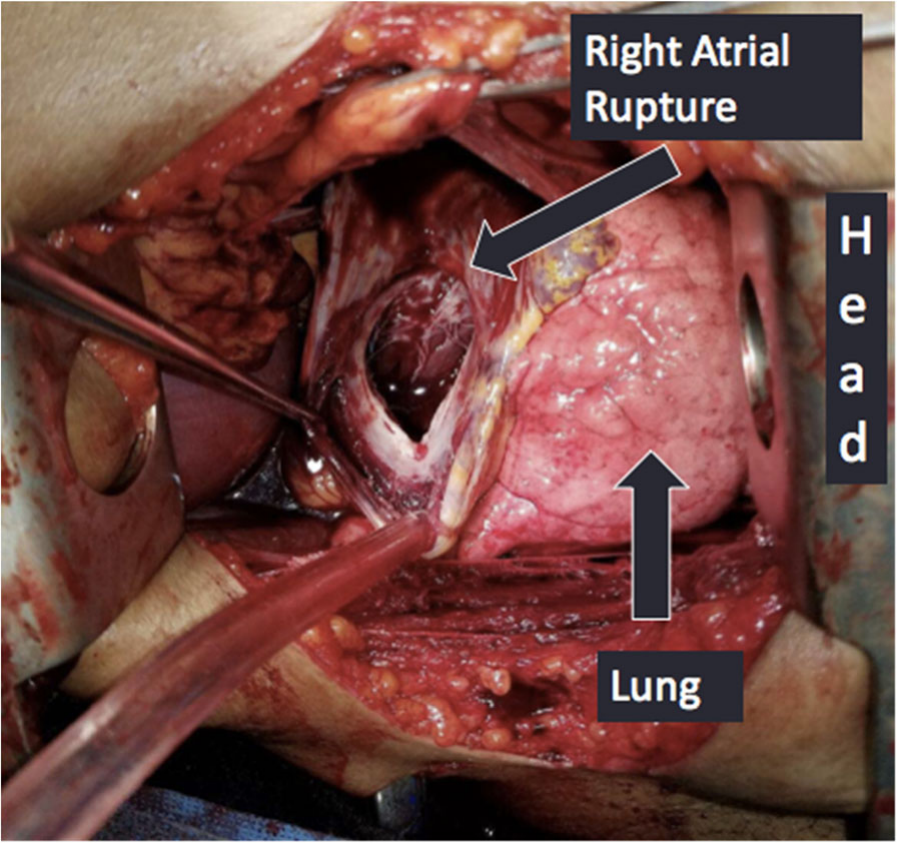
Case 1: Pericardial and right atrial rupture

A left anterolateral thoracotomy incision was made with the patient in a supine position. There was a significant amount of blood upon entry into the chest cavity, and there was no cardiac tamponade. Despite cross clamping the pulmonary hilum, the bleeding continued. While being resuscitated with the massive transfusion protocol, there was no end tidal CO2 noted. The pericardium was opened, heart delivered, and cardiac massage was started. A separate defect in the superior, right side of the pericardium was found as well as a 3 cm defect in the anterior right atrium (See Fig. 2). Despite measures to control hemorrhage and resuscitate the patient, the patient did not survive. The blood loss was greater than 6 l.

## Case Report Two

The second case is a 58-year-old male who was involved in a high-speed MVC. The patient had a prolonged extrication from his vehicle and was intubated after being found unresponsive. On arrival his GCS was 3 T. He was hypotensive with FAST negative for intra-abdominal blood, but a large left hemothorax was identified on the left upper quadrant view. A chest tube was placed with initially 900 mL of blood out followed by 200 mL per hour for 2 h. Based on the output and ongoing resuscitation requirements, the decision was made to take the patient to the OR.

**Fig. 3:**
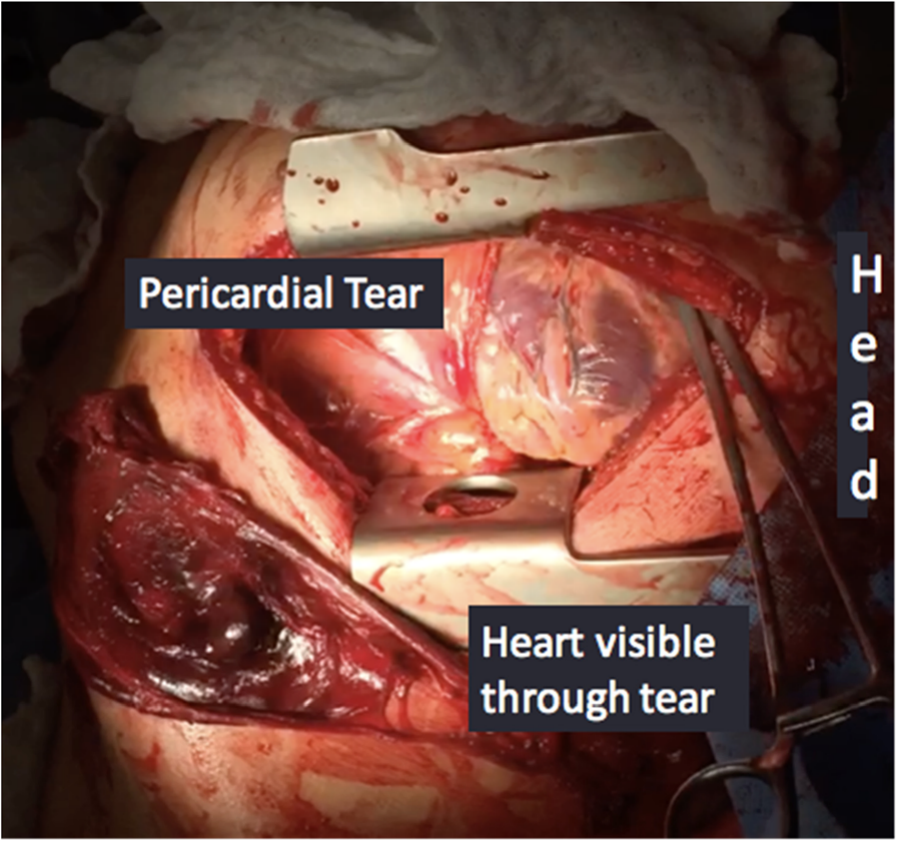
Case 2: Pericardial tear with left atrial appendage laceration

A left anterolateral thoracotomy incision was made with the patient in supine position. There was approximately 1 L of clotted blood within the chest cavity coming from an anterior pericardial laceration, about 15 cm in length. Through the defect there was brisk bleeding coming from a 1 cm laceration on the left atrial appendage (See Fig. 3). The injury was debrided and repaired using a running 3–0 polypropylene suture over a Satinsky clamp. The pericardial defect was closed to prevent cardiac herniation. The patient recovered well and was eventually discharged home.

## Discussion

In our case series, we present two patients who were taken to the OR with uncontained blunt cardiac rupture. With a 89.2% mortality rate for chamber rupture [7], most of these patients do not make it to the hospital, let alone the OR. With such a high mortality rate, we feel it is important to review these cases to discuss their presentations and treatment options.

The first patient arrived unresponsive, tachycardic and hypotensive after blunt trauma. Following Advanced Trauma Life Support (ATLS) principles, the patient was intubated and resuscitation started. The FAST exam was performed, and failed to show any pericardial effusion. This was due to the rupture not only of the right atrium, but the pericardium itself. This allowed exsanguination into the pleural space. From autopsy data, fatal blunt cardiac trauma with chamber rupture occurs most often to the left ventricle [6, 10]. In contrast, in patients that present to the hospital, right atrial rupture is more common. These injuries are seen at the superior vena cava-atrial junction, inferior vena cava-atrial junction or in right atrial appendage (a common site of rupture due to its thin wall) [6, 10].

Pericardial tears often occur secondary to increased intra-abdominal pressure or lateral decelerative forces from either side. In blunt injury with cardiac rupture, 70% of the time the pericardium stays intact; in 30% of cases it ruptures [12]. Cardiac herniation with cardiac dysfunction can occur in conjunction with these tears. The heart may be displaced into either the pleural cavity or even into the abdomen depending on the location of the pericardial defect. In right-sided pericardial rupture, the heart can twist along the caval axis, preventing venous return, and leading to the surprising discovery of an empty pericardial cavity during a resuscitative left anterolateral thoracotomy [1].

Alternatively, patients may bleed into the mediastinum or into the pleural cavity. This often leads to a delay in diagnosis. This is not surprising, as most pericardial ruptures will be diagnosed with thoracotomies in the OR [8]. May et al. (1999) describes two similar cases in which the patients had cardiac and pericardial ruptures. Ultimately, the patients succumbed to their injuries due to exsanguination into the mediastinum and thorax [11]. This unfortunately, was the same result as our first case.

The second patient presented with left sided hemothorax after a MVC. The differential of left sided hemothorax after blunt injury is broad and ranges from intercostal artery injury or lung parenchyma laceration to great vessel or cardiac injury. Cardiac rupture is a rare etiology of hemothorax. In a recent series of cases, Oizumi et al. [12] describes only 11 known patients who have survived blunt cardiac rupture with a concomitant pericardial defect [12]. Our patient would perhaps be the 12th.

Desforges and coauthors reported the first repair of a blunt myocardial rupture in 1955, successfully closing a right atrial perforation secondary to a MVC [13]. The traditional approach for a left-sided hemothorax is a left anterolateral thoracotomy. To improve access to the heart, the incision can be extended with to a median sternotomy or a right anterolateral thoracotomy incision. In situations where primarily a cardiac injury is suspected, it would be appropriate to start with a median sternotomy. A left thoracotomy often does not provide adequate exposure to the heart or ascending aorta.

For cardiac lacerations, cardiorrhaphy can be accomplished via several techniques. Methods to temporize bleeding to allow time for definitive repair include the use of a foley balloon through the defect to occlude the opening or temporary closure with direct pressure, staplers or vascular clamps [1]. Definitive repair with double armed monofilament pledgeted suture repair either an interrupted or running technique has been described [9, 14]. If large septal defects, uncontrollable bleeding or coronary artery injury is identified intraoperatively, consultation with a cardiothoracic surgeon is recommended. The patient may need cardiopulmonary bypass or extracorporeal life support to allow for definitive repair. If the patient is able to survive the initial insult, heart failure may ensue requiring assist devices or even transplant [9].

## Conclusion

In summary, we present two cases of uncontained atrial rupture from blunt cardiac trauma. Contained ruptures with an intact pericardium can present as a cardiac tamponade while uncontained ruptures present with hemomediastinum or hemothorax and are more likely to be fatal. A high degree of suspicion is required to rapidly diagnose and perform the cardiorrhaphy to offer the best chance at survival.

## Abbreviations

ATLS: Advanced Trauma Life Support
FAST: Focused assessment with sonography for trauma
GCS: Glasgow Coma Scale
MVC: Motor vehicle collisions
OR: Operating room
